# Characterization and comparative genomics analysis of RepA_N multi-resistance plasmids carrying *optrA* from *Enterococcus faecalis*

**DOI:** 10.3389/fmicb.2022.991352

**Published:** 2023-01-27

**Authors:** Enbao Zhang, Shuaizhou Zong, Wei Zhou, Jinzhi Zhou, Jianzhong Han, Daofeng Qu

**Affiliations:** ^1^Key Laboratory of Food Quality and Safety, School of Food Science and Biotechnology, Zhejiang Gongshang University, Hangzhou, China; ^2^Zhejiang Provincial Center for Animal Disease Prevention and Control, Hangzhou, China

**Keywords:** plasmid, antibiotic resistance, *optrA*, MDR, mobile elements

## Abstract

**Introduction:**

This research aimed to investigate the antibiotic resistance of *Enterococcus faecalis* from swine farms in Zhejiang Province and the prevalence and transmission mechanism of oxazolidone resistance gene *optrA*.

**Method:**

A total of 226 *Enterococcus faecalis* were isolated and their resistance to 14 antibiotics was detected by broth microdilution. The resistance genes were detected by PCR.

**Results:**

The antibiotic resistance rate of 226 isolates to nearly 57% (8/14) of commonly used antibiotics was higher than 50%. The resistance rate of tiamulin was highest (98.23%), that of tilmicosin, erythromycin, tetracycline and florfenicol was higher than 80%, and that of oxazolidone antibiotic linezolid was 38.49%. The overall antibiotics resistance in Hangzhou, Quzhou and Jinhua was more serious than that in the coastal cities of Ningbo and Wenzhou. The result of PCR showed that *optrA* was the main oxazolidinone and phenicols resistance gene, with a detection rate of 71.68%, and *optrA* often coexisted with *fexA* in the isolates. Through multi-locus sequence typing, conjugation transfer, and replicon typing experiments, it was found that the horizontal transmission mediated by RepA_N plasmid was the main mechanism of *optrA* resistance gene transmission in E. faecalis from Zhejiang Province. Two conjugative multi-resistance plasmids carrying *optrA*, RepA_N plasmid pHZ318-*optrA* from Hangzhou and Rep3 plasmid from Ningbo, were sequenced and analyzed. pHZ318-*optrA* contain two multidrug resistance regions (MDR), which contributed to the MDR profile of the strains. *optrA* and *fexA* resistance genes coexisted in IS*1216*E-*fexA*-*optrA*-*ferr*-*erm*(A)-IS*1216*E complex transposon, and there was a partial sequence of Tn*554* transposon downstream. However, pNB304-optrA only contain *optrA*, *fexA* and an insertion sequence IS*Vlu1*. The presence of mobile genetic elements at the boundaries can possibly facilitate transfer among *Enterococcus* through inter-replicon gene transfer.

**Discussion:**

This study can provide theoretical basis for ensuring the quality and safety of food of animal origin, and provide scientific guidance for slowing down the development of multi-antibiotic resistant *Enterococcus*.

## 1. Introduction

The livestock and poultry breeding industry in China is gradually changing from decentralized breeding to intensive breeding, and antibiotics are widely used as growth promoters and treatment antibiotics for bacterial diseases (Wang et al., 2021). The unreasonable use of antibiotics leads to the enhancement of bacterial antibiotic resistance and the diffusion of antibiotic resistance genes, which brings great harm to public health (Sun, 2014). *Enterococcus faecalis* is one of the common pathogens causing infectious diseases in humans or animals, and oxazolidinone antibiotics are the “last line of defense” in the treatment of vancomycin-resistant *Enterococcus*, so monitoring its antibiotic resistance is of great health significance (Senior, 2000; Yasufuku et al., 2011). However, there are few studies on the antibiotic resistance of *Enterococcus faecalis* from swine in Zhejiang Province. Previous studies mainly focused on clinical *Enterococcus* or gram-negative bacteria such as *Escherichia coli* and *Salmonella* from swine (Falagas et al., 2011; Anjum et al., 2016).

Linezolid is the first class of oxazolidinones, which is a fully synthetic antibiotic that binds to the initial complex and inhibits protein synthesis (Bender et al., 2018). Linezolid is active against a wide range of Gram-positive bacteria, including methicillin-resistant *Staphylococcus aureus* (MRSA) and Vancomycin-resistant *Enterococcus* (VRE), and has been the “last line of defense” against gram-positive multidrug-resistant bacteria following vancomycin (Carmen et al., 2018). Most bacteria, including *Enterococcus*, have multiple copies of genes encoding 23S rRNA, and the level of resistance to linezolid expression depends on the number of these genes containing relevant mutations (Pillai et al., 2002). Currently, three different groups of acquired resistance genes which can product cross-resistance to linezolid and florfenicol have been identified. These genes include *cfr*, *optrA*, and *poxtA* (Liu et al., 2012; Yang et al., 2015; Hao et al., 2019). *cfr* resistance gene modifies A2503 methyltransferase in bacterial 23S rRNA, and was first identified in *Staphylococcus sciuri* of animal origin (Schwarz et al., 2000). The gene *optrA* encodes a ribosomal protection protein of the ABC-F family, and imparts transferable resistance to oxazolidone (linezolid and tedizolid) and phenicols (chloramphenicol and florfenicol; Wang et al., 2015). *poxtA* also encodes a ribosomal protection protein of the ABC-F family and has recently been identified in MRSA (Antonelli et al., 2018).

Oxazolidinones antibiotics are not allowed to be used in foodborne animal farming, however, linezolid-resistant *Enterococcus* carrying *optrA* gene in transferable plasmids have been detected in these animals (Carmen et al., 2018). This gene coexists with antibiotic resistance genes of commonly used animal antibiotics (penicillins, tetracyclines, lincosamides, and aminoglycosides), indicating their role in co-selection of multidrug resistant bacteria, which poses a risk to public health (Carmen et al., 2018). Although oxazolidinones are not used in the swine industry, florfenicol is widely used to treat bacterial infections in swine, which may promote the spread of *cfr*, *optrA*, and *poxtA* genes in swine farms (Zhao et al., 2016). Moreover, it is still unknown how frequently *optrA* gene occurs in the *Enterococcus* in Zhejiang Province, and the number of related multi-resistance plasmids containing *optrA* is not very high in the NCBI database. Moreover, the genetic background of *optrA* also needs to be further studied.

In this study, the antibiotic resistance of the 14 common antibiotics in *Enterococcus faecalis* from swine farms was determined. This research provided resistance profile of *Enterococcus faecalis* and comparison analysis of multiple antibiotic resistance from swine farms in five cities of Zhejiang Province. In addition, the presence, transferability and related mobile elements of the *optrA* gene in *Enterococcus* of swine origin was investigated. Furthermore, two conjunctive plasmids harboring *optrA* were analyzed to clarify the basis for dissemination. This research enriched the study of plasmids containing *optrA* resistance genes in the NCBI database.

## 2. Materials and methods

### 2.1. Bacterial strains

A total of 226 non-duplicate *Enterococcus faecalis* isolates were collected from 15 swine farms in Hangzhou, Ningbo, Jinhua, Quzhou and Wenzhou of Zhejiang Province, China in 2020. A list of collected fecal samples from swine was presented in Table 1. Colonies of red to purple growing on *Enterococcus* Chromogenic medium were initially considered *Enterococcus* strains. The isolates were further identified as *Enterococcus faecalis* by VITEK-2 Compact and 16S rRNA gene identification using the universal 16 s primers 27F and 1492R (Lane, 1991). *Enterococcus faecalis* JH2-2 served as the recipient strain in conjugal transfer experiments. JH2-2 was resistant to rifampicin and fusidic acid, but sensitive to other antibiotics.

**Table 1:** The results of *Enterococcus* isolation from fecal samples in Zhejiang.

Source	Number of samples	Number of isolates	Isolated rate (%)
Hangzhou	105	41	39.05
Ningbo	100	18	18.00
Jinhua	120	68	56.67
Quzhou	100	37	37.00
Wenzhou	120	62	51.67
Total	545	226	41.47

### 2.2. Antimicrobial susceptibility testing

Antibiotic susceptibility testing of *Enterococcus faecalis* strains was performed by broth microdilution method (MIC) and classified as resistant, intermediate, or susceptible according to the CLSI criteria for *Enterococcus* as the breakpoints were shown in Supplementary Table S2. *Enterococcus faecalis* strains exhibiting resistance to three or more classes of antimicrobials were considered to be multi-drug resistant (MDR). *Enterococcus faecalis* ATCC 29212 served as the control strain.

### 2.3. PCR analysis

*Enterococcus faecalis* strains were screened for the presence of the antibiotic resistance genes and for the type of plasmid replicon by PCR using the primers listed in Supplementary Table S3 [*cfr, cfr(B), optrA, poxtA, fexA, fexB, floR, cmlA and lsa*(E)] and Supplementary Table S4 (including 10 plasmid *rep* genes, such as pIP501, pRE25, pS86), as described previously (Jensen et al., 2010). PCR amplification consisted of denaturation at 95°C for 5 min followed by denaturation at 94°C for 30 s, annealing at their respective annealing temperature for 30 s, and polymerization at 72°C for 40 s for a total of 30 cycles, and a final extension at 72°C for 10 min. All the PCR products were subjected to Sanger sequencing.

### 2.4. Multilocus sequencing typing

Multilocus sequencing typing (MLST) of 30 oxazolidone resistance gene positive *Enterococcus faecalis* strains isolated from five cities in Zhejiang province was studied by PCR method. Seven pair primers of housekeeping genes of *Enterococcus faecalis* were searched from pubmlst website^1^ and synthesized by Shanghai Biological Engineering Co., Ltd. (Supplementary Table S5). The PCR amplification condition was as described previously (Ruiz-Garbajosa et al., 2006). The positive PCR products were sent to biological companies for sequencing, and the sequences were submitted to *Enterococcus* MLST database^2^ to analyze the housekeeping genes number and the Sequence type of each strain was determined.

### 2.5. Conjugal transfer experiments

Conjugal transfer experiments were performed with rifampin and fusidic acid resistant *Enterococcus faecalis* JH2-2 used as a recipient and each of the 20 *optrA*-positive *Enterococcus faecalis* isolates as a donor. Brain Heart Infusion (BHI) was supplemented with 64 μg/ml rifampicin, 64 μg/ml fusidic acid and 16 μg/ml florfenicol to screen transconjugants. After incubation at 37°C for 24 h, colonies growing on these selective plates were further confirmed by antimicrobial sensitivity experiments and VITEK-2 Compact.

### 2.6. Sequencing and analysis

The two conjugatable plasmids of the transconjugants HZ318-JH2-2 and NB304-JH2-2 were sequenced by the Illumina MiSeq platforms. The two plasmids DNA was extracted from the transconjugants HZ318-JH2-2 and NB304-JH2-2 using the Qiagen Large-Construct Kit. After extraction, the mate-pair library was constructed. Then MiSeq (Illumina, CA, United States)was used to sequence the plasmids, and Newbler 2.6 was used to assemble the illumina read sets straight off the MiSeq. Quality control and removing low-quality data were performed with TrimMomatic 0.36 (Bolger Anthony et al., 2014). Gapfiller V1.11 is used to fill gaps (Nederbragt, 2014). Finally, Cytoscape was used to spline the sequence to obtain the final cyclized plasmids. The plasmid sequences were submitted to Rast, Genemarks, Glimmer, and Prodigal library for preliminary gene prediction then submitted to ISFinder, Integrall, ResFinder, and TN Number Registry for mobile elements (Boetzer and Pirovano, 2012). Gene organization diagrams were drawn by running a gene alignment program in Perl language (Supplementary material) with Ubuntu 18.04 LTS^3^ and Inkscape 0.48.1.^4^

### 2.7. Determination of plasmid stability

The *optrA* positive transconjugants HZ318-JH2-2, NB304-JH2-2 and the control strain JH2-2 were inoculated into BHI plate and cultured in a constant temperature incubator at 37°C for 12~18 h, and a single colony of each strain was inoculated into 5 ml LB broth medium for 12 h. Absorb 5 μl overnight culture solution and add sterilized BHI broth to 5 ml, dilute it by 1,000 times, then subculture for 14 days. The bacterial solution was diluted by 10 times gradient series, and 100 μl suitable diluted bacterial solution was coated on LB plate without antibiotics for overnight culture. Transfer the single colony on the plate to LB-florfenicol resistant plate and non-resistant LB plate, respectively. Plasmid retention ratio = number of single colonies on florfenicol resistant plate/number of single colonies on non-resistant plate. The stability curve of plasmid was drawn with the retention ratio (%) of plasmid in *Enterococcus faecalis* as ordinate and the passage days (d) as abscissa.

## 3. Results

### 3.1. Analysis of antibiotic resistance of *Enterococcus faecalis*

**Figure 1:**
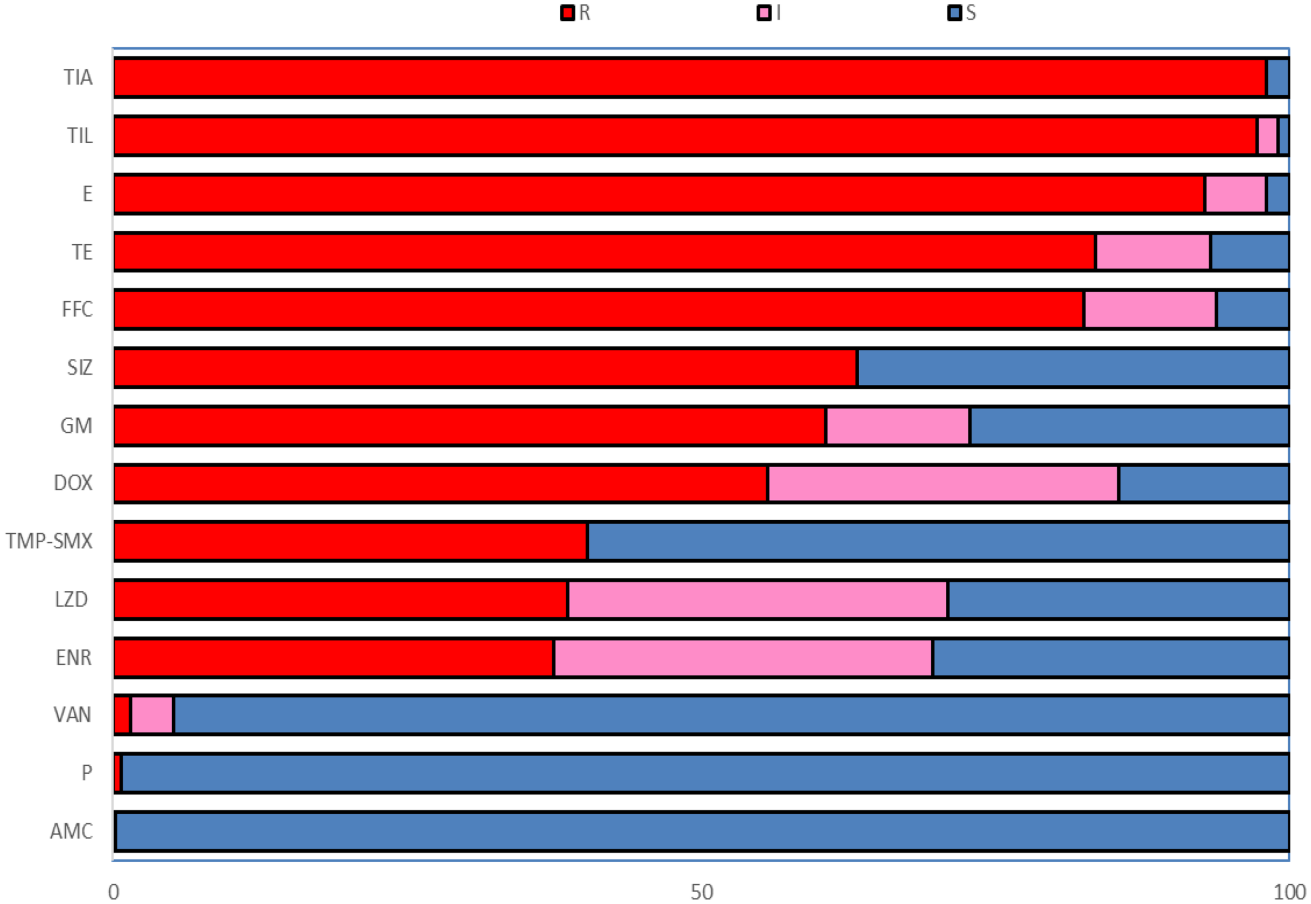
The resistance rate to 14 antibiotics of *Enterococcus faecalis* from swine. R, Resistant; I, Intermediate; S, Susceptible; P, Penicillin; AMC, Amoxicillin/clavulanic acid; GM, Gentamicin; E, Erythromycin; TIL, Tilmicosin; FFC, Florfenicol; SIZ, Sulfisoxazole; TMP-SME, Trimethoprim/Sulfamethoxazole; VAN, Vancomycin; DOX, Doxycycline; TE, Tetracycline; ENR, Enrofloxacin; LZD, Linezolid; TIA, Tiamulin.

In this study, 226 strains of *Enterococcus faecalis* were identified by VITEK-2 Compact and the amplification of 16S rRNA gene (Supplementary Table S6). Table 1 showed the isolation of *Enterococcus faecalis* in Hangzhou, Ningbo, Jinhua, Quzhou and Wenzhou of Zhejiang Province, in which the highest isolation rate of *Enterococcus faecalis* was 56.67% in Jinhua, and the lowest in Ningbo was 18%. It can be seen from Figure 1 that among the 14 antibiotics, 226 strains of *Enterococcus faecalis* isolated from swine developed strong resistance to nearly 57% of the commonly used antibiotics, their resistance rates were all higher than 50%, and maintained high sensitivity to penicillin, vancomycin and amoxicillin/clavulanic acid. Strains resistant to amoxicillin/clavulanic acid (AMC) were not detected, but producing varying degrees of resistance to the other 13 antibiotics.

**Figure 2:**
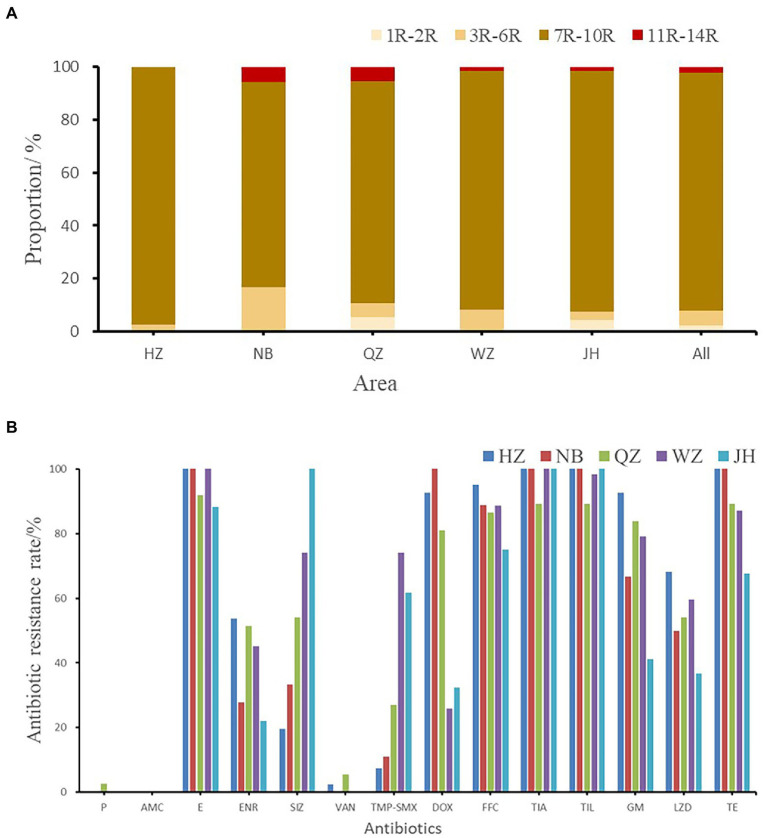
**(A)** The multi-drug resistance of *Enterococcus faecalis* from swine in Zhejiang. **(B)** The resistance rate of *E. faecalis* from five cities. HZ, Hangzhou; NB, Ningbo; QZ, Quzhou; WZ, Wenzhou; JH, Jinhua; All, Zhejiang Province.

The resistance rates of 226 *Enterococcus faecalis* isolates to tiamulin, tilmicosin and erythromycin were 98.23%, 97.78%, and 92.92%, respectively, and the resistance rates to tetracycline and florfenicol were 83.50% and 82.51%, which were all higher than 80%. *Enterococcus faecalis* produced resistance to 1 to 11 antibiotics of these 14 antibiotics, and no strains resistant to 12 or more antibiotics were found. As can be seen from Figure 2, the antibiotic resistance grade of *Enterococcus faecalis* is concentrated in 7R~10R, accounting for 90%, respectively, and the proportion of multiple antibiotic resistant bacteria (3R–14R) is as high as 97.79%.

### 3.2. Comparison of multiple antibiotic resistance in five cities

As shown in Figure 2B, the antibiotic resistance characteristics of *Enterococcus faecalis* isolates from different regions were compared. It was found that the isolates in each region were highly resistant to erythromycin, florfenicol, tiamulin, tilmicosin, and tetracycline, and some of them were even 100% resistant. *Enterococcus faecalis* in Hangzhou, Quzhou and Wenzhou were completely resistant to erythromycin, while *Enterococcus faecalis* in Hangzhou, Ningbo and Jinhua were completely resistant to tiamulin and tilmicosin. Hangzhou and Ningbo are completely resistant to tetracycline.

There were significant differences in antibiotic resistance rates of enrofloxacin, sulfamethoxazole, trimethoprim/sulfamethoxazole, gentamicin and linezolid antibiotics in five different cities (*p* < 0.05). One strain of *Enterococcus faecalis* resistant to penicillin was found in Quzhou, and one strain and two strains of *Enterococcus faecalis* resistant to vancomycin were found in Hangzhou and Quzhou, respectively.

### 3.3. Detection of the resistance genes in *Enterococcus faecalis strains*

**Figure 3:**
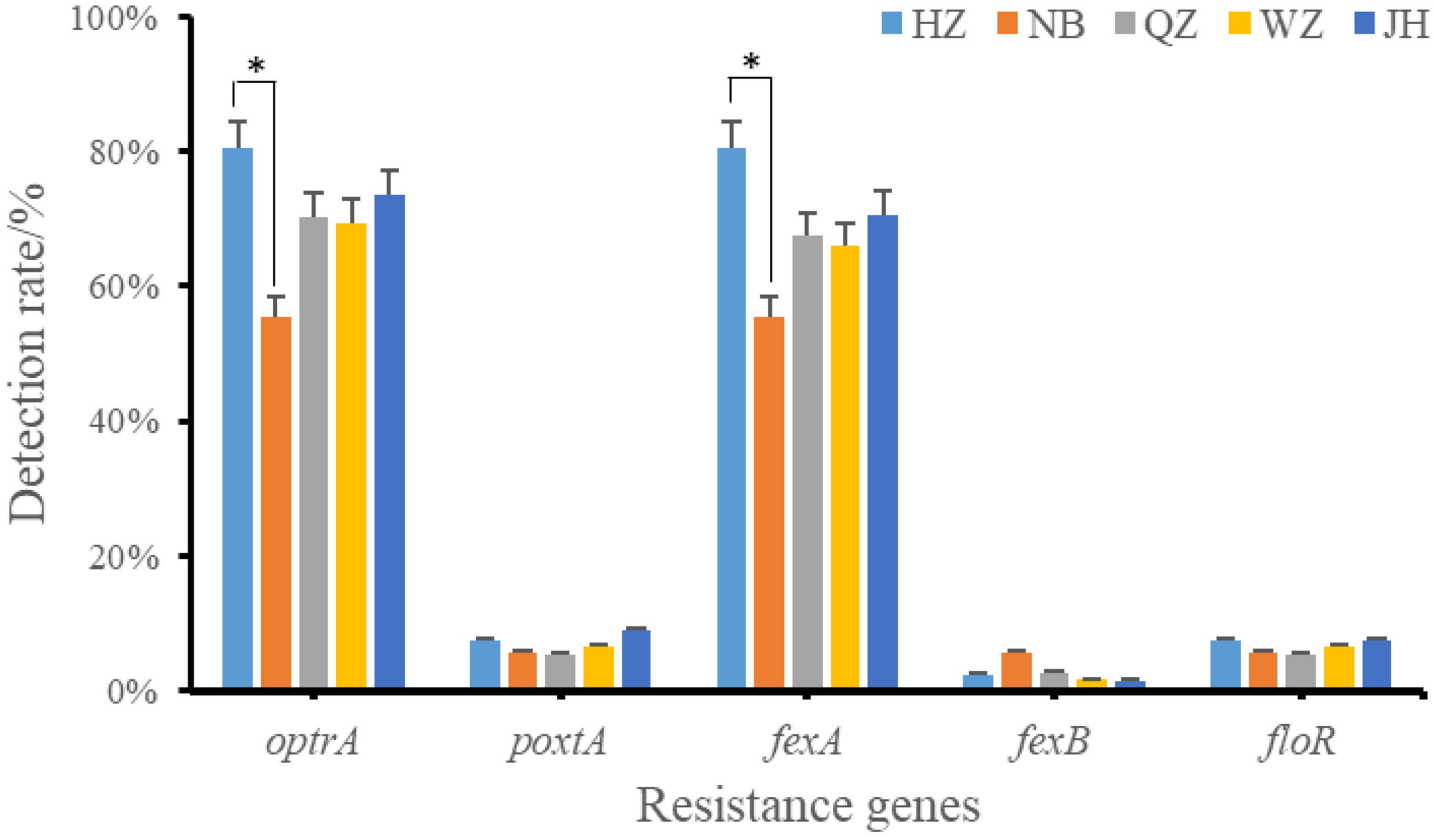
Positive rate of oxazolidinones and phenicoles resistance genes. *indicates that there is a significant difference between the two groups. HZ, Hangzhou; NB, Ningbo; QZ, Quzhou; WZ, Wenzhou; JH, Jinhua.

The resistance genes of oxazolidinone, phenicols, and lincoamine-pleuromutilin-streptomycin A were searched in 226 resistant strains. 38.49% (87/226) strains was resistant to oxazolidinone and 82.30% (186/226) was resistant to phenicols. The results showed that only two oxazolidinone resistance genes were detected in these antibiotic resistant strains, including *optrA* and *poxtA* (Figure 3). A total of 173 strains contained oxazolidinones resistance genes (*optrA* and *poxtA*). The detection rates of *optrA* and *poxtA* were 71.68% (162/226) and 7.08% (16/226), respectively. The detection rates of *optrA* in Hangzhou, Ningbo, Quzhou, Wenzhou and Jinhua were 80.49% (33/41), 55.56% (10/18), 70.27% (26/37), 69.35% (43/62), and 73.53% (50/68), respectively. The detection rate of *optrA* in Hangzhou was significantly higher than that in Ningbo (*p* < 0.05). *optrA* and *fexA* were the main phenicols resistance genes, and the detection rates were 71.68% (162/226) and 69.47% (157/226), respectively. A total of 121 strains of *Enterococcus faecalis* containing lincoamine-pleuromutilin-streptomycin A resistance gene *lsa*(E) were detected, with a detection rate of 53.54% (121/226).

### 3.4. MLST analysis of *Enterococcus faecalis*

Through MLST analysis of 30 strains of oxazolidinone resistance gene positive *Enterococcus faecalis*, the evolution and genetic relationship among different strains were studied, and the transmission mechanism of *optrA* resistance gene in *Enterococcus faecalis* was inferred. The results showed that the distribution of different strains of *Enterococcus faecalis* was scattered. There were 19 different types of *Enterococcus faecalis* in 30 strains, of which 6 strains were dominant ST714 type, 3 strains were ST16 type, and the rest ST types contained 1 or 2 strains of *Enterococcus faecalis* (Supplementary Table S6). The ST type of each strain was shown in Supplementary Table S6. There are a small number of strains of *Enterococcus faecalis* with same ST type, but the ST types of most strains are different, indicating that horizontal transmission may be the main mechanism of the prevalence of oxazolidinone resistance gene *optrA* in *Enterococcus faecalis*.

### 3.5. The *optrA* gene is transferable

The transconjugants isolated by mating *optrA* positive strains with JH2-2 were resistant to rifampicin and florfenicol antibiotics. 20 *optrA* positive strains were conjugated and transferred successfully, which indicated that some of the *optrA* genes were located on plasmids and could be transmitted by conjugation transfer. The highest conjugation transfer efficiency of HZ318 was 5.8 × 10–4, and the lowest conjugation efficiency was NB304, which was only 6.8 × 10^−6^. According to the MIC results of wild strains and transconjugant, the resistant plasmid has been successfully introduced into the recipient bacteria (Supplementary Table S7). Among the 12 isolates of *Enterococcus faecalis*, 7 strains were RepA-N type plasmid with replicon type of Rep 9 family, 3 strains were lnc18 type plasmid with replicon type of rep2 family, and 2 strains were Rep_3 Small_theta type plasmid with replicon type of rep18 and repUS40, respectively. The results showed that the horizontal transmission of oxazolidone resistance gene *optrA* may be dominated by RepA_N type plasmid in swine farms of Zhejiang Province.

### 3.6. Characterization of plasmids pHZ318-optrA and pNB304-optrA

**Figure 4:**
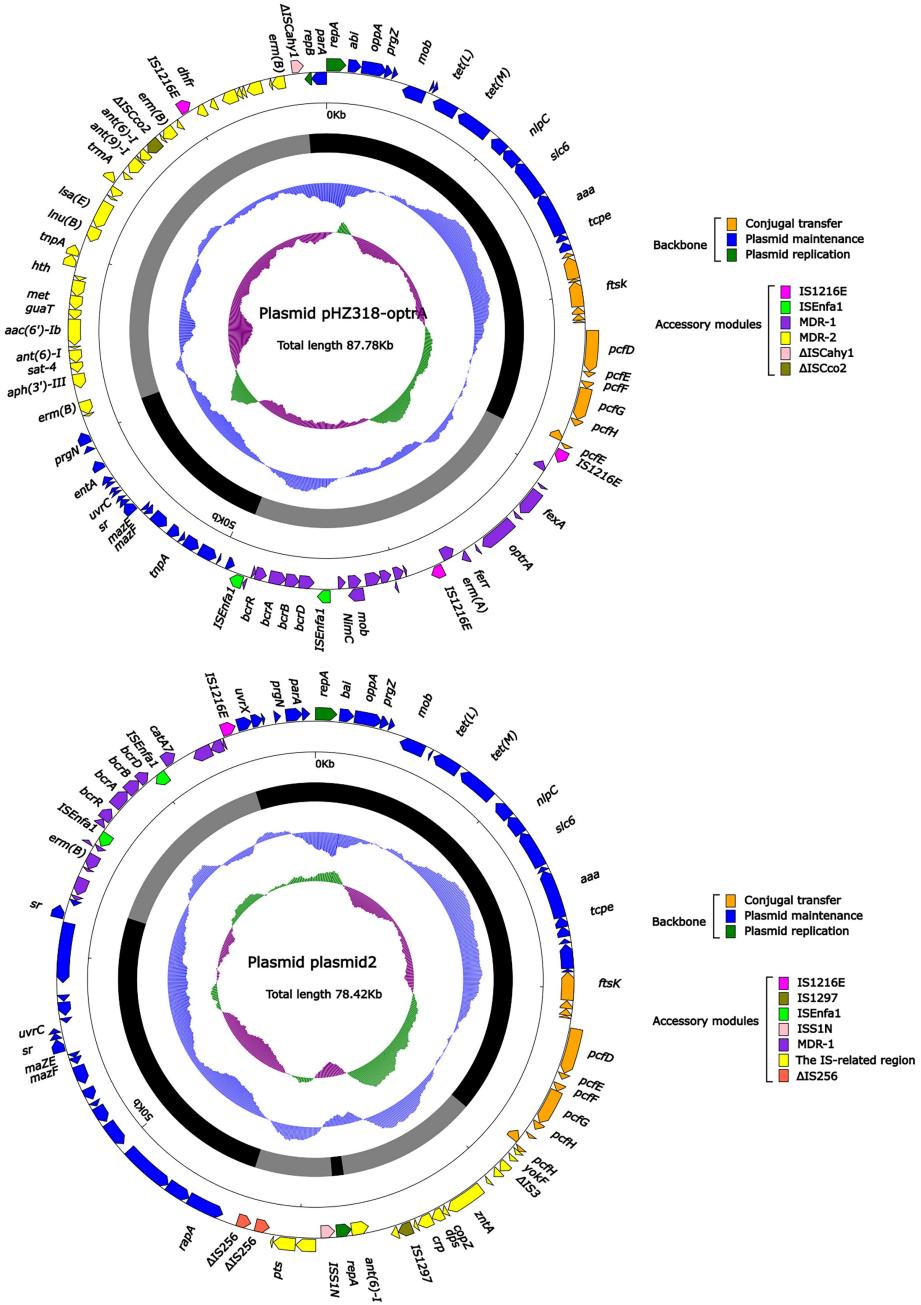
The complete sequence diagram of pHZ318-optrA and plasmid2. In the figure, the innermost ring represents (G − C)/(G + C); the blue ring indicates GC content, and the outward depression indicates that the GC content is higher than the mean value. Black area in the outer circle represents the backbone area and gray area represents the accessory modules. The outermost circle shows the distribution of genes represented by colored arrows in the plasmid.

**Figure 5:**
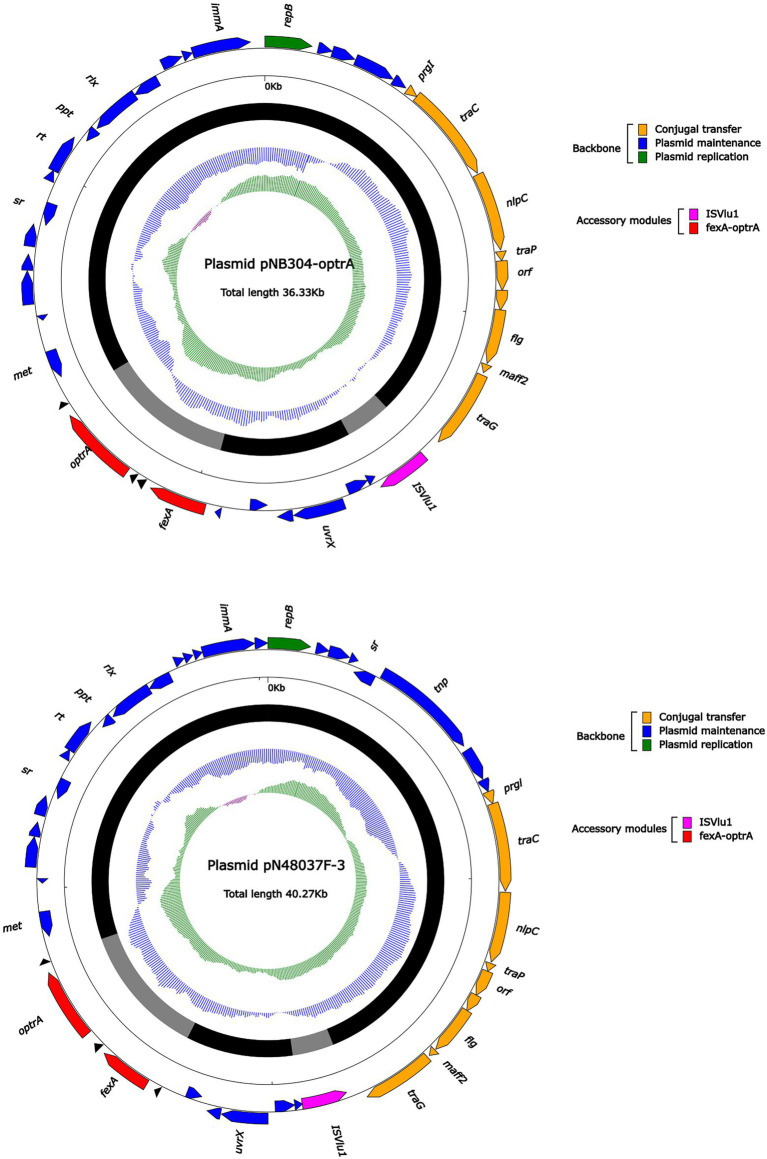
The complete sequence diagram of pNB304-optrA and N48037F-3.

**Figure 6:**
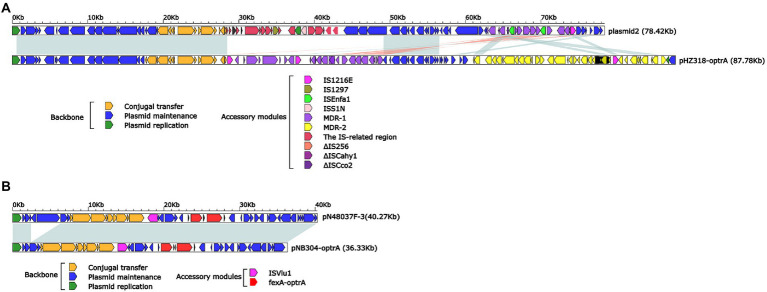
**(A)** Linear comparison of pHZ318-optrA and plasmid2. **(B)** Linear comparison of pNB304-optrA and pN48037F-3.

**Figure 7:**
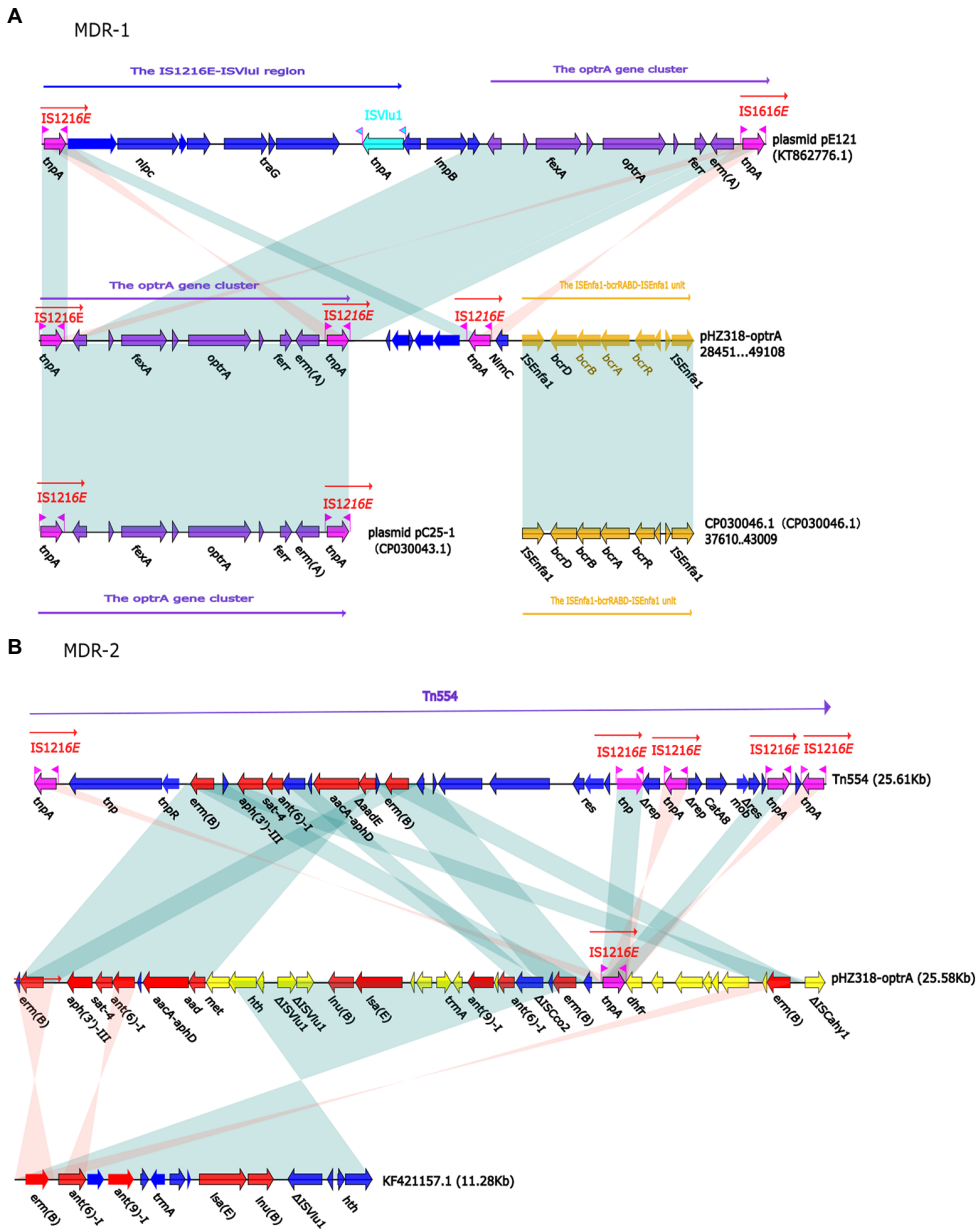
**(A)** The organization and alignment of MDR-1 region of pHZ318-optrA. **(B)** The organization and alignment of MDR-2 region of pHZ318-optrA. Genes are indicated by arrows. Genes, mobile elements, and other features are colored based on functional classification. Shadows indicate nucleotide homology is greater than 95%. The number in parentheses indicates the location of the fragment in the plasmid.

Plasmid pHZ318-optrA belonged to RepA_N plasmid with a total length of 87,785 bp, and 112 ORFs (Table 2; Figure 4). The replication initiation proteins of pHZ318-optrA belong to rep9 family, contains replicons *repA* and *repB*. Plasmid2 was also RepA_N and the backbone sequence was highly similar to pHZ318-optrA, so chosen as a reference (Figure 5). The entire backbone area of the plasmid pHZ318-optrA was inserted by MDR-1 and MDR-2, while that of plasmid2 was interrupted by a multidrug resistance region MDR-1 and *ant* (6)-I related region with multiple IS sequences (Figure 6). The MDR-1 region of pHZ318-optrA consisted of the following mobile elements: IS*1216*E-*fexA*-*optrA*-*ferr*-*erm*(A)-IS*1216*E, IS*1216*E and The IS*Enfa1*-*bcr*RABD-IS*Enfa1* unit (Figure 7). IS*1216*E was a complete insertion sequence with a full length of 808 bp, flanked by 24 bp reverse repeats and interspersed with 681 bp IS*6* family transposable enzymes. Two IS*1216*E and *fexA*-*optrA*-*ferr*-*erm*(A) gene clusters together formed a transposable unit, which was found to be identical to the transposition unit in plasmidpc25-1 (CP030043.1) through comparison. Multidrug resistance region MDR-2 was mainly composed of some intermediate sequences of Tn*554* transposon, IS insertion sequence and antibiotic resistance gene clusters including erm (B) and *lsa* (E).

**Table 2:** Major features of plasmids analyzed.

Type	Plasmid Name			
	pHZ318-optrA	plasmid2	pNB304-optrA	N48037F-3
Sequence Type	RepA_N	RepA_N	Rep3	Rep3
The total length(kb)	87,785	78,419	36,331	40,269
The number of ORF	112	93	40	48
Average GC content (%)	34.53	34.77	34.34	34.01
Accessorymodules	MDR-1 region and MDR-2 region	MDR-1 region and *ant* (6)-I related region	*fexA*-*optrA* and IS*Vlu1*	*fexA*-*optrA* and IS*Vlu1*

Plasmid pNB304-optrA belonged to Rep3 plasmid with a total length of 36,331 bp and 40 ORFs. The plasmid also contained two accessory modules, comprising *fexA*-*optrA* and IS*Vlu1* regions, without no other transposons and mobile elements. The antibiotic resistance gene only contain aminol resistance gene *fexA* and oxazolidinone resistance gene *optrA*. The main difference of plasmid pNB304-optrA and the reference plasmid N48037F-3 (Genbank Number: CP028723.1) was that pNB304-optrA lost part of the backbone area due to specific recombination in the downstream of *sr* gene after the initiation of plasmid replication.

**Figure 8:**
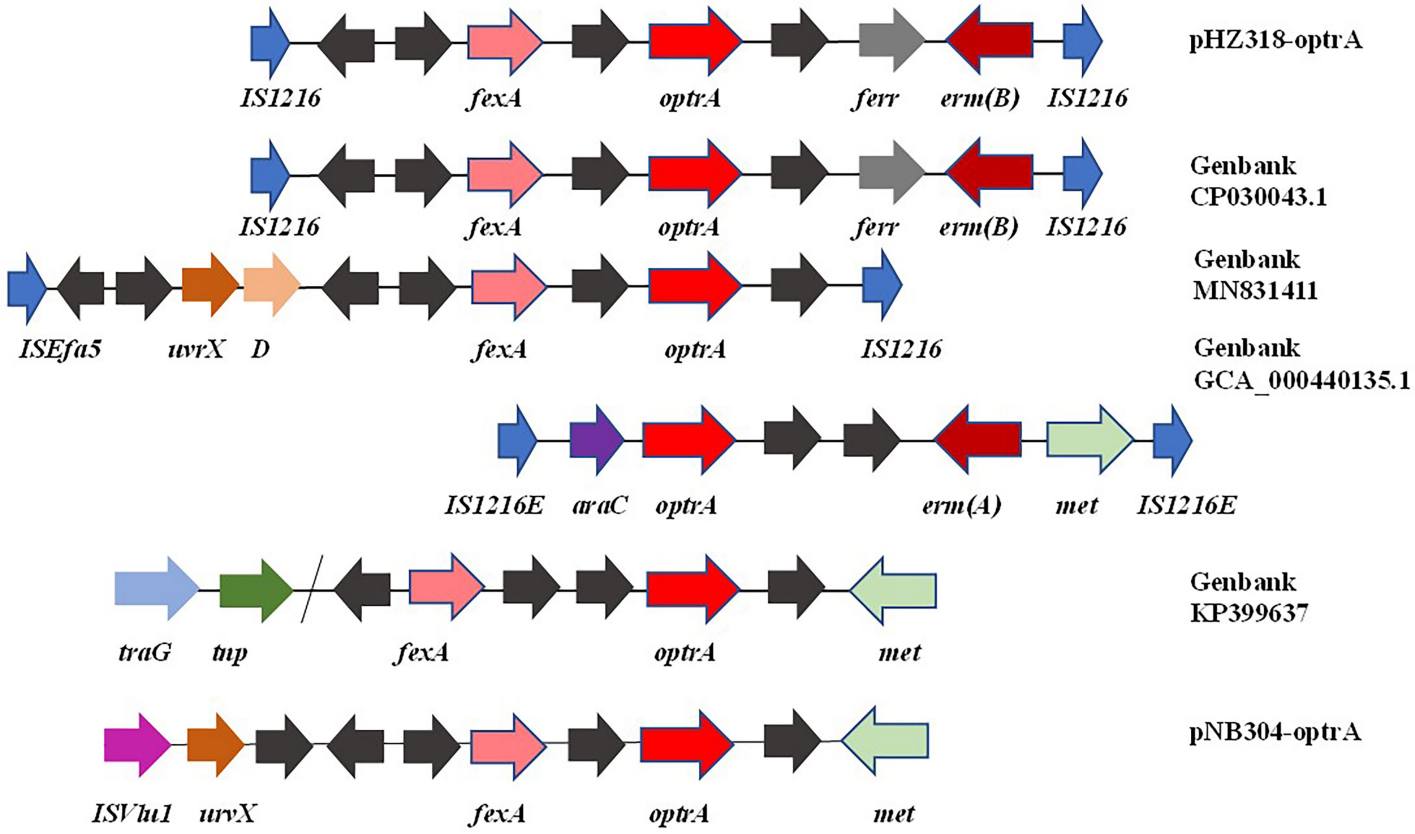
Diversity of flanking genetic environment of *optrA* gene on plasmids.

By comparing the flanking sequences of *optrA* in pHZ318-optrA and pNB304-optrA, it was found that there was a phenicols resistance gene *fexA* in the upstream of *optrA* and a macrolide resistance gene *erm*(B) in the downstream of *optrA* in pHZ318-optrA. Figure 8 showed the flanking genetic environment of *optrA* in different strains, indicating genetic diversity. The plasmid-mediated oxazolidone resistance gene *optrA* was mostly related to IS insertion sequence, especially IS*1216E* mobile element. Other researchers also found similar genetic background IS*1216*E-*fexA*-*optrA*-*erm*(A)-IS*1216*E in many different *optrA* vectors, suggesting that IS*1216*E may promote the common transfer of *optrA*, *fexA*, and *erm* (A) among different plasmids.

### 3.7. Stability analysis of *optrA* resistant plasmids

**Figure 9:**
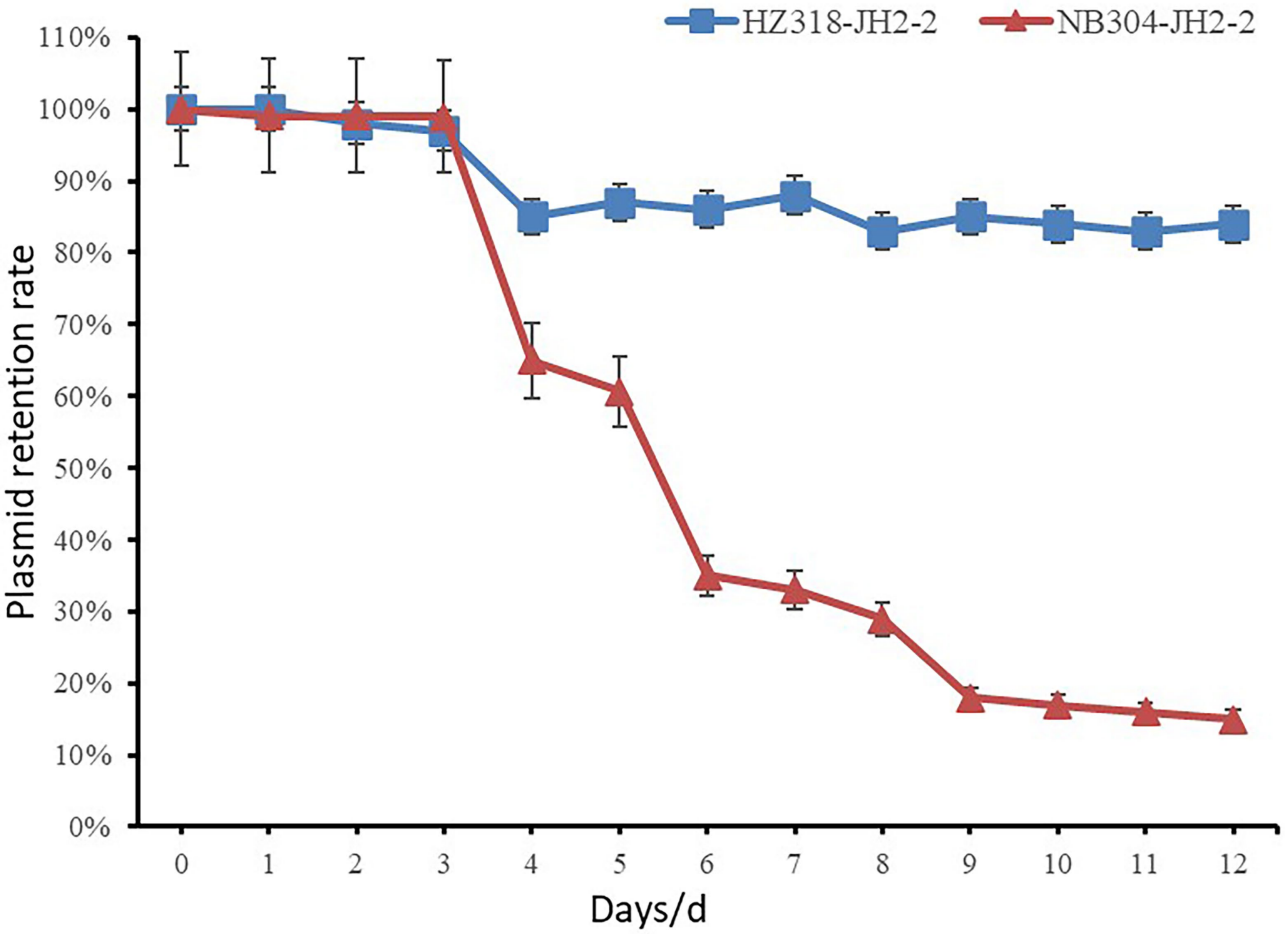
Stability of the plasmids in conjugants.

The plasmids stability of transconjugants of HZ318 and NB304 strains without antibiotic selection pressure was analyzed. Results as shown in Figure 9, a small part of plasmid were lost in transconjugant HZ318-JH2-2 under no antibiotic selection pressure, while most of plasmids were lost in transconjugant NB304-JH2-2, in which plasmid HZ318-JH2-2 was lost by 16.3% ± 1.1% in 12 days. NB304-JH2-2 plasmid was lost by 85.0% ± 1.2% in 12 days. This indicated that there was some difference in the stability of the two plasmids.

## 4. Discussion

In this study, 226 *Enterococcus faecalis* strains isolated from swine farms in Zhejiang Province showed high-level resistance to nearly 57% (8/14) of commonly used antibiotics, and their antibiotic resistance rates were all higher than 50%. This showed that the traditional antibiotics were no longer suitable for the treatment of bacterial diseases in swine farms, especially tiamulin, which was widely used in livestock and poultry breeding as veterinary antibiotic and feed additives. More importantly, the resistance rate of linezolid, known as the “last line of defense” of Gram-positive bacteria, was as high as 38.49% (87/226), and the intermediary rate of antibiotic resistance was 32.30% (73/226). In this study, it was found that the antibiotic resistance of strains resistant to linezolid was often more serious. According to multiple antibiotic resistance analysis, it was basically concentrated in 7 or more multiple antibiotic resistance strains.

In this study, the resistance rate of *Enterococcus faecalis* to florfenicol was 82.51%, which was higher than 16.1% in Tibet (Peng et al., 2014). This may be directly related to the antibiotic use level of swine in Tibetan. Swine in Tibetan are generally in the form of stocking or semi-stocking, and the antibiotic use level is relatively low, but the swine industry is more developed in Zhejiang, the antibiotic use level is higher, resulting in the selection pressure of bacterial antibiotic resistance. In view of the high antibiotic resistance of linezolid, oxazolidinone antibiotic resistance genes *cfr*, *cfr*(B), *optrA*, and *poxtA* were detected in this study. *optrA* resistance gene made strains resistant to linezolid and also produced cross-resistance to phenicols antibiotics. Through further detection of other phenicols resistance genes *fexA*, *fexB*, *cmlA*, and *floR*, it was found that *optrA* + *fexA* genotype was the main mechanism of resistance of *Enterococcus* to oxazolidone and phenicols antibiotics. The emergence of *optrA* multidrug resistance gene and its prevalence in *Enterococcus* have weakened the efficacy of linezolid in the treatment of clinical antibiotic resistant strains to a certain extent, posing a huge potential threat to human health.

Insertion sequence, transposon and integron can assist the horizontal transfer of antibiotic resistance genes among bacteria, which is an important reason for the widespread existence of antibiotic resistance genes in different environments and microorganisms (Stokes and Gillings, 2011). Plasmids are important vectors for carrying these Mobile Genetic Elements (MGEs) and acquired antibiotic resistance genes, and these MGEs provide a mechanism for intracellular movement, so that antibiotic resistance genes have the opportunity to “escape” to host chromosomes or other plasmids (Partridge et al., 2018). In the presence of antibiotic selection pressure, the interaction between different types of MGEs promotes the evolution of a variety of antibiotic resistant bacteria (Partridge et al., 2010).

We identified two conjunctive multi-resistance plasmids carrying *optrA* and analyzed the genetic context of *optrA* and MDR region. IS*1216*E could be reassembled to form a circular intermediate, which could integrate *optrA* into the plasmid or chromosome and spread among different bacterial species. In addition, there was a partial complete sequence of Tn*554* transposon downstream of the plasmid *optrA*, which endows the strain with resistance to macrolides and streptomycin. And studies have shown that IS*1216*E, Tn*554* and Tn*558* may promote the horizontal transmission of *optrA* and *poxtA* (Kang et al., 2019). The existence of other antibiotic resistance genes, including *fexA*, *fexB* and *erm*(A), may lead to the common selection of *optrA* and *poxtA*, which makes it widely spread in *Enterococcus*.

IS*1216*E has also been reported in previous literature, such as vancomycin resistance gene *VanA* in *Enterococcus* and multidrug resistance genes *poxtA*, *optrA*, and *cfr* in *Enterococcus* and *Staphylococci* (Darini et al., 2000; Liu et al., 2013; Antonelli et al., 2018). This shows that IS*1216*E plays an important role in the transmission of antibiotic resistance genes in Gram-positive bacteria. In this experiment, a partial complete sequence of transposon Tn*554* was also found downstream of IS*1216*E-*fexA*-*optrA*-*ferr*-*erm*(A)-IS*1216*E transposon of plasmid pHZ318-optrA. We should pay more attention to the transmission of transferable oxazolidinone resistance genes in foodborne Gram-positive bacteria, because livestock and poultry farms may become a repository of transferable oxazolidinone resistance genes, and these antibiotic resistance genes may be transmitted to humans through the food chain.

## 5. Conclusion

The presence of multiple mobile elements in a *optrA*-carrying multi-resistance plasmid makes it flexible. These elements aid its persistence and dissemination among *Enterococcus faecalis* and potentially other Gram-positive bacteria. This study demonstrated the abundance of oxazolidinone and phenicols resistance genes of swine origin in Zhejiang Province. It also upgraded the research on plasmids containing *optrA* antibiotic resistance genes. This research also provided the antibiotic profile of bacteria isolated from swine farms in Zhejiang Province, indicating that local farmers can possibly reduce the use of specific antibiotics to reduce the evolution of multi-resistant strains.

## Data availability statement

The data presented in the study are deposited in the NCBI repository, accession numbers OQ181208 and OQ181209.

## Author contributions

EZ: visualization, data curation, software, and writing-original draft preparation. SZ: conceptualization, methodology, formal analysis, and writing-original draft preparation. WZ: visualization and investigation. JZ: software and writing—review and editing. JH: formal analysis and resources. DQ: conceptualization, resources, supervision, and writing-review and editing. All authors contributed to the article and approved the submitted version.

## Funding

This work was supported by National Natural Science Foundation of China (no. 32172188) and the Key R&D projects in Zhejiang Province (nos. 2018C02024 and 2020C02031).

## Conflict of interest

The authors declare that the research was conducted without any commercial or financial relationships that could be construed as a potential conflict of interest.

## Publisher’s note

All claims expressed in this article are solely those of the authors and do not necessarily represent those of their affiliated organizations, or those of the publisher, the editors and the reviewers. Any product that may be evaluated in this article, or claim that may be made by its manufacturer, is not guaranteed or endorsed by the publisher.

## Footnotes

^1^ http://www.mlst.net

^2^ https://pubmlst.org/organisms/

^3^ https://ubuntu.com/

^4^ https://inkscape.org/en/
