# Supplementary material for: Characterization and comparative genomics analysis of RepA_N multi-resistance plasmids carrying optrA from Enterococcus faecalis
Source: Front Microbiol. 2023 Jan 27;13:991352. doi: 10.3389/fmicb.2022.991352 (PMC9911807; doi:10.3389/fmicb.2022.991352)
Supplement: Supplementary file 1 [file Table_1.docx]

***Supplementary Material***

**Table S1 The information of resistance profile of *Enterococcus faecalis* isolates**

| Isolates | P | AMC | E | ENR | SIZ | VAN | TMP-SMX | DOX | FFC | TIA | TIL | GM | LZD | TE |
| --- | --- | --- | --- | --- | --- | --- | --- | --- | --- | --- | --- | --- | --- | --- |
| HZZ1-04 | S | S | R | R | R | S | S | R | R | R | R | S | S | R |
| HZZ1-07 | S | S | R | R | R | S | S | I | R | R | R | R | I | R |
| HZZ1-08 | S | S | R | R | S | S | S | R | I | R | R | R | S | R |
| HZZ1-09 | S | S | R | S | S | S | S | R | R | R | R | R | I | R |
| HZZ1-13 | S | S | R | R | R | S | R | R | R | R | R | S | S | R |
| HZZ2-02 | S | S | R | S | S | S | S | R | R | R | R | R | R | R |
| HZZ2-04 | S | S | R | R | S | S | S | R | R | R | R | R | R | R |
| HZZ2-05 | S | S | R | R | S | S | S | R | R | R | R | R | R | R |
| HZZ2-06 | S | S | R | R | S | S | S | R | R | R | R | R | R | R |
| HZZ2-07 | S | S | R | R | R | S | S | R | R | R | R | R | S | R |
| HZZ2-08 | S | S | R | R | S | S | S | R | R | R | R | R | R | R |
| HZZ2-09 | S | S | R | R | S | S | S | R | R | R | R | R | R | R |
| HZZ2-12 | S | S | R | R | S | S | S | R | R | R | R | R | R | R |
| HZZ2-14 | S | S | R | R | S | S | S | R | R | R | R | R | R | R |
| HZZ2-15 | S | S | R | R | S | S | S | R | R | R | R | R | R | R |
| HZZ2-16 | S | S | R | R | S | S | S | R | R | R | R | R | R | R |
| HZZ2-17 | S | S | R | R | S | S | S | R | S | R | R | R | I | R |
| HZZ2-18 | S | S | R | R | S | S | S | R | R | R | R | R | R | R |
| HZZ2-19 | S | S | R | R | S | S | S | R | R | R | R | R | R | R |
| HZZ2-20 | S | S | R | R | S | S | S | R | R | R | R | R | R | R |
| HZZ2-21 | S | I | R | R | S | S | S | R | R | R | R | R | R | R |
| HZZ2-22 | S | S | R | R | S | S | S | R | R | R | R | R | R | R |
| HZZ3-01 | S | S | R | I | R | S | S | R | R | R | R | R | R | R |
| HZZ3-03 | S | S | R | S | S | S | S | R | R | R | R | R | I | R |
| HZZ3-04 | S | S | R | S | S | S | S | R | R | R | R | R | R | R |
| HZZ3-06 | S | S | R | S | S | S | S | R | R | R | R | R | R | R |
| HZZ3-07 | S | S | R | S | S | S | S | R | R | R | R | R | I | R |
| HZZ3-08 | S | S | R | I | S | S | S | R | R | R | R | R | R | R |
| HZZ3-10 | S | S | R | I | R | S | S | R | R | R | R | R | R | R |
| HZZ3-11 | S | S | R | S | S | S | S | S | R | R | R | S | I | R |
| HZZ3-12 | S | S | R | S | S | S | S | R | R | R | R | R | R | R |
| HZZ3-13 | S | S | R | S | S | S | S | R | R | R | R | R | R | R |
| HZZ3-14 | S | S | R | I | R | S | S | R | R | R | R | R | R | R |
| HZZ3-15 | S | S | R | S | S | S | S | R | R | R | R | R | R | R |
| HZZ3-16 | S | S | R | S | S | S | S | R | R | R | R | R | I | R |
| HZZ3-17 | S | S | R | S | S | S | S | R | R | R | R | R | I | R |
| HZZ3-18 | S | S | R | S | S | R | R | S | R | R | R | R | R | R |
| HZZ4-06 | S | S | R | S | S | R | R | R | R | R | R | R | R | R |
| HZZ4-10 | S | S | R | R | S | S | S | R | R | R | R | R | S | R |
| HZZ5-09 | S | S | R | S | R | S | S | R | R | R | R | R | R | R |
| HZZ5-14 | S | S | R | R | S | S | S | R | R | R | R | R | R | R |
| NBZ1-02 | S | S | R | R | R | S | S | R | R | R | R | I | R | R |
| NBZ1-10 | S | S | R | I | R | S | S | R | R | R | R | R | R | R |
| NBZ1-11 | S | S | R | R | S | S | S | R | R | R | R | I | R | R |
| NBZ1-12 | S | S | R | R | S | S | S | R | R | R | R | I | R | R |
| NBZ1-13 | S | S | R | R | S | S | S | R | R | R | R | I | R | R |
| NBZ5-01 | S | S | R | S | S | S | S | R | R | R | R | R | I | R |
| NBZ5-02 | S | S | R | S | S | S | S | R | I | R | R | R | S | R |
| NBZ5-04 | S | S | R | I | S | S | R | R | R | R | R | R | S | R |
| NBZ5-05 | S | S | R | S | S | S | S | R | R | R | R | R | R | R |
| NBZ5-06 | S | S | R | S | R | S | S | R | R | R | R | R | R | R |
| NBZ5-08 | S | S | R | I | S | S | S | R | R | R | R | R | S | R |
| NBZ5-09 | S | S | R | I | R | S | S | R | R | R | R | R | R | R |
| NBZ5-13 | S | S | R | S | S | S | S | R | R | R | R | R | I | R |
| NBZ5-14 | S | S | R | R | R | S | S | R | I | R | R | R | S | R |
| NBZ5-16 | S | S | R | S | R | S | R | R | R | R | R | R | R | R |
| NBZ5-18 | S | S | R | I | S | S | S | R | R | R | R | I | S | R |
| NBZ5-19 | S | S | R | I | S | S | S | R | R | R | R | I | S | R |
| NBZ5-20 | S | S | R | S | S | S | S | R | R | R | R | R | I | R |
| QZZ1-02 | S | S | R | S | R | S | R | R | R | R | R | R | I | R |
| QZZ1-03 | S | S | R | S | R | S | S | R | R | R | R | R | R | R |
| QZZ1-04 | S | S | R | R | R | I | S | R | R | R | R | R | S | R |
| QZZ1-05 | S | S | R | R | R | S | R | R | R | R | R | R | R | R |
| QZZ1-06 | S | S | R | S | S | S | S | R | R | R | R | R | R | R |
| QZZ1-10 | S | S | R | S | R | S | S | R | R | R | R | R | R | R |
| QZZ1-13 | R | S | R | I | R | S | R | R | R | R | R | R | I | R |
| QZZ1-15 | S | S | R | I | R | S | R | R | R | R | R | R | I | R |
| QZZ1-17 | S | S | R | R | S | S | S | S | R | R | R | I | R | R |
| QZZ1-18 | S | S | R | S | R | S | S | R | R | R | R | R | S | R |
| QZZ1-19 | S | S | R | R | S | R | R | S | R | S | R | R | S | R |
| QZZ2-03 | S | S | R | I | S | S | S | R | R | R | R | S | R | R |
| QZZ2-05 | S | S | R | I | S | S | S | R | R | R | R | I | S | R |
| QZZ2-08 | S | S | R | R | S | I | R | R | R | S | R | R | S | R |
| QZZ2-09 | S | S | R | R | R | R | S | R | R | R | R | R | R | R |
| QZZ2-11 | S | S | R | R | S | S | R | R | R | S | R | R | R | R |
| QZZ2-16 | S | S | R | S | R | S | R | R | R | R | R | R | R | R |
| QZZ3-01 | S | S | R | S | R | S | S | R | R | R | R | R | I | R |
| QZZ3-03 | S | S | R | S | S | I | S | R | R | R | R | R | S | R |
| QZZ3-04 | S | S | S | R | R | S | S | R | R | R | I | I | R | R |
| QZZ3-07 | S | S | R | I | R | S | R | I | S | R | R | R | I | I |
| QZZ3-11 | S | S | R | R | R | S | S | R | R | R | R | R | R | R |
| QZZ4-01 | S | S | R | R | R | S | S | R | R | R | R | R | R | R |
| QZZ4-02 | S | S | I | S | S | S | S | S | S | R | I | S | I | S |
| QZZ4-03 | S | S | R | R | S | S | R | R | R | S | R | R | S | R |
| QZZ4-04 | S | S | R | R | R | S | S | I | R | R | I | R | R | S |
| QZZ4-05 | S | S | R | R | S | S | S | S | R | R | R | R | I | R |
| QZZ4-06 | S | S | R | R | S | S | S | R | S | R | R | R | S | R |
| QZZ4-07 | S | S | I | S | S | S | S | I | I | R | S | I | I | S |
| QZZ4-10 | S | S | R | I | R | S | S | R | R | R | R | R | R | R |
| QZZ5-01 | S | S | R | S | R | S | S | R | R | R | R | R | R | R |
| QZZ5-02 | S | S | R | R | R | S | S | R | R | R | R | R | R | R |
| QZZ5-04 | S | S | R | R | S | S | S | R | R | R | R | R | R | R |
| QZZ5-06 | S | S | R | S | S | S | S | R | R | R | R | R | R | R |
| QZZ5-08 | S | S | R | R | S | S | S | R | R | R | R | R | R | R |
| QZZ5-09 | S | S | R | R | S | S | S | R | S | R | R | R | I | R |
| QZZ5-10 | S | S | R | R | R | S | S | R | R | R | R | R | R | R |
| JHZ1-01 | S | S | R | R | R | S | R | R | R | R | R | S | S | R |
| JHZ1-02 | S | S | R | S | R | S | R | R | S | R | R | R | R | R |
| JHZ1-03 | S | S | S | I | R | S | S | S | R | R | R | I | R | S |
| JHZ1-04 | S | S | R | S | R | S | R | S | R | R | R | R | S | R |
| JHZ1-05 | S | S | R | S | R | S | S | R | I | R | R | S | R | S |
| JHZ1-06 | S | S | R | I | R | S | R | S | R | R | R | I | S | R |
| JHZ1-08 | S | S | R | R | R | S | S | R | R | R | R | R | R | R |
| JHZ1-09 | S | S | R | I | R | S | R | S | S | R | R | R | I | R |
| JHZ1-10 | S | S | S | S | R | S | S | R | R | R | R | S | S | R |
| JHZ1-12 | S | S | R | S | R | S | R | S | S | R | R | R | R | S |
| JHZ1-13 | S | S | R | R | R | I | S | S | R | R | R | S | S | R |
| JHZ1-14 | S | S | R | S | R | S | R | R | R | R | R | R | I | R |
| JHZ2-01 | S | S | R | S | R | S | S | S | R | R | R | I | R | R |
| JHZ2-02 | S | S | R | S | R | S | R | I | S | R | R | R | S | R |
| JHZ2-03 | S | S | R | S | R | S | S | S | R | R | R | R | R | R |
| JHZ2-05 | S | S | R | R | R | S | R | R | R | R | R | S | R | S |
| JHZ2-06 | S | S | R | I | R | S | R | R | S | R | R | R | I | R |
| JHZ2-07 | S | S | S | S | R | S | S | S | R | R | R | S | S | R |
| JHZ2-08 | S | S | R | I | R | S | S | R | R | R | R | R | R | S |
| JHZ2-09 | S | S | R | I | R | S | R | S | S | R | R | I | S | R |
| JHZ2-10 | S | S | R | S | R | S | R | I | R | R | R | R | I | R |
| JHZ2-11 | S | S | S | I | R | S | R | S | I | R | R | S | R | R |
| JHZ2-12 | S | S | R | S | R | S | S | R | S | R | R | I | R | R |
| JHZ2-13 | S | S | R | I | R | S | R | S | R | R | R | R | S | R |
| JHZ2-14 | S | S | R | R | R | S | S | R | R | R | R | R | R | S |
| JHZ3-01 | S | S | R | S | R | I | R | S | S | R | R | S | I | R |
| JHZ3-02 | S | S | R | S | R | S | R | S | R | R | R | R | R | S |
| JHZ3-03 | S | S | R | I | R | S | S | S | R | R | R | S | S | R |
| JHZ3-04 | S | S | S | R | R | S | R | R | S | R | R | I | R | R |
| JHZ3-05 | S | S | R | S | R | S | R | S | R | R | R | R | S | R |
| JHZ3-07 | S | S | R | I | R | S | S | S | S | R | R | S | I | R |
| JHZ3-08 | S | S | R | S | R | S | S | I | R | R | R | S | I | R |
| JHZ3-09 | S | S | R | R | R | S | R | S | R | R | R | R | R | S |
| JHZ3-10 | S | S | R | S | R | S | R | R | I | R | R | R | S | R |
| JHZ3-11 | S | S | R | S | R | S | S | S | R | R | R | S | R | R |
| JHZ3-12 | S | S | R | I | R | S | R | S | R | R | R | S | S | R |
| JHZ3-14 | S | S | R | R | R | S | R | S | S | R | R | S | S | R |
| JHZ3-15 | S | S | R | S | R | S | R | R | R | R | R | R | I | S |
| JHZ4-01 | S | S | R | I | R | S | S | S | R | R | R | S | I | R |
| JHZ4-02 | S | S | S | S | R | S | R | S | R | R | R | R | S | R |
| JHZ4-03 | S | S | R | R | R | S | R | S | R | R | R | S | R | I |
| JHZ4-04 | S | S | R | S | R | S | R | R | R | R | R | S | S | S |
| JHZ4-05 | S | S | R | R | R | S | S | S | R | R | R | S | S | S |
| JHZ4-06 | S | S | S | S | R | S | R | S | R | R | R | R | S | S |
| JHZ4-07 | S | S | R | I | R | S | S | S | R | R | R | S | I | R |
| JHZ4-08 | S | S | R | S | R | S | R | S | R | R | R | R | S | R |
| JHZ4-09 | S | S | R | R | R | S | S | R | R | R | R | S | R | R |
| JHZ4-10 | S | S | R | S | R | S | R | S | S | R | R | S | S | S |
| JHZ4-11 | S | S | R | I | R | S | R | S | R | R | R | R | S | R |
| JHZ4-12 | S | S | R | I | R | S | S | S | R | R | R | S | I | S |
| JHZ4-13 | S | S | I | S | R | S | S | R | R | R | R | S | S | S |
| JHZ4-15 | S | S | R | R | R | S | R | S | R | R | R | S | R | R |
| JHZ4-16 | S | S | R | S | R | S | R | R | R | R | R | R | S | R |
| JHZ4-17 | S | S | R | S | R | S | S | S | R | R | R | S | I | R |
| JHZ4-18 | S | S | R | R | R | S | S | S | R | R | R | I | R | S |
| JHZ4-19 | S | S | R | R | R | S | R | R | R | R | R | R | S | R |
| JHZ4-20 | S | S | R | S | R | S | R | S | R | R | R | S | R | R |
| JHZ4-21 | S | S | R | S | R | S | R | S | S | R | R | S | S | R |
| JHZ5-01 | S | S | R | S | R | S | S | S | R | R | R | R | R | R |
| JHZ5-02 | S | S | R | R | R | S | R | S | R | R | R | S | S | S |
| JHZ5-03 | S | S | R | S | R | S | R | R | R | R | R | S | R | S |
| JHZ5-04 | S | S | R | S | R | S | R | S | R | R | R | R | I | R |
| JHZ5-06 | S | S | R | S | R | S | S | S | R | R | R | S | S | R |
| JHZ5-07 | S | S | R | S | R | S | R | R | R | R | R | R | S | S |
| JHZ5-08 | S | S | R | S | R | S | R | S | R | R | R | S | R | R |
| JHZ5-09 | S | S | R | I | R | S | R | S | R | R | R | S | S | R |
| JHZ5-10 | S | S | R | S | R | S | S | R | S | R | R | S | R | S |
| JHZ5-11 | S | S | R | S | R | S | R | S | R | R | R | R | I | S |
| WZZ1-01 | S | S | R | R | R | S | R | S | R | R | R | R | S | R |
| WZZ1-02 | S | S | R | S | R | S | S | R | R | R | R | R | R | R |
| WZZ1-04 | S | S | R | I | S | S | R | S | S | R | R | R | I | R |
| WZZ1-05 | S | S | R | R | R | S | R | S | R | R | R | S | R | S |
| WZZ1-06 | S | S | R | S | R | S | R | R | R | R | R | R | R | R |
| WZZ1-07 | S | S | R | R | R | S | S | S | R | R | R | R | R | R |
| WZZ1-09 | S | S | R | I | R | I | R | S | R | R | R | S | I | R |
| WZZ1-10 | S | S | R | S | R | S | S | R | R | R | R | R | R | R |
| WZZ1-11 | S | S | R | R | S | S | R | S | S | R | R | R | R | R |
| WZZ2-01 | S | S | R | R | R | S | R | S | R | R | R | R | I | R |
| WZZ2-02 | S | S | R | I | R | S | R | I | R | R | R | R | R | R |
| WZZ2-04 | S | S | R | R | R | S | R | S | R | R | S | I | S | S |
| WZZ2-05 | S | S | R | S | S | S | R | R | R | R | R | R | R | R |
| WZZ2-06 | S | S | R | S | R | S | S | S | R | R | R | R | I | S |
| WZZ2-07 | S | S | R | R | R | S | R | R | R | R | R | R | R | R |
| WZZ2-08 | S | S | R | S | R | S | S | S | R | R | R | R | R | R |
| WZZ2-09 | S | S | R | R | S | S | R | S | R | R | R | S | R | R |
| WZZ2-11 | S | S | R | R | R | I | R | R | R | R | R | R | I | R |
| WZZ2-12 | S | S | R | R | S | S | R | S | R | R | R | R | R | R |
| WZZ2-13 | S | S | R | I | R | S | S | S | R | R | R | R | R | R |
| WZZ2-14 | S | S | R | S | R | S | R | S | I | R | R | R | R | I |
| WZZ2-16 | S | S | R | R | S | S | R | I | R | R | R | R | I | R |
| WZZ3-01 | S | S | R | R | R | S | R | R | R | R | R | R | R | R |
| WZZ3-02 | S | S | R | S | R | S | R | S | R | R | R | I | R | R |
| WZZ3-03 | S | S | R | R | R | S | R | S | R | R | R | R | R | R |
| WZZ3-04 | S | S | R | I | S | S | R | R | R | R | R | R | S | R |
| WZZ3-05 | S | S | R | I | R | S | S | S | R | R | R | S | R | R |
| WZZ3-07 | S | S | R | R | R | S | R | S | R | R | R | R | I | R |
| WZZ3-08 | S | S | R | S | R | S | S | S | R | R | R | R | R | R |
| WZZ3-09 | S | S | R | I | S | S | R | S | R | R | R | R | S | R |
| WZZ3-10 | S | S | R | R | R | S | S | S | R | R | R | I | R | R |
| WZZ3-11 | S | S | R | S | R | S | R | R | R | R | R | R | R | S |
| WZZ3-12 | S | S | R | I | R | S | R | S | I | R | R | R | I | R |
| WZZ3-13 | S | S | R | R | R | S | S | R | R | R | R | R | R | R |
| WZZ3-14 | S | S | R | S | R | S | R | S | R | R | R | R | R | R |
| WZZ4-01 | S | S | R | R | R | S | R | I | S | R | R | R | S | R |
| WZZ4-03 | S | S | R | I | S | S | S | S | R | R | R | R | S | R |
| WZZ4-04 | S | S | R | R | R | S | R | R | R | R | R | I | R | R |
| WZZ4-05 | S | S | R | R | R | S | R | S | R | R | R | R | I | R |
| WZZ4-06 | S | S | R | S | R | S | R | S | R | R | R | S | R | S |
| WZZ4-07 | S | S | R | I | R | S | R | S | R | R | R | R | S | R |
| WZZ4-08 | S | S | R | R | S | S | S | R | R | R | R | R | R | R |
| WZZ4-10 | S | S | R | S | R | S | R | S | R | R | R | R | I | R |
| WZZ4-11 | S | S | R | S | R | S | S | S | R | R | R | R | R | R |
| WZZ4-12 | S | S | R | R | R | S | R | S | R | R | R | R | I | R |
| WZZ4-13 | S | S | R | S | S | S | R | R | R | R | R | R | R | R |
| WZZ4-14 | S | S | R | S | R | S | R | S | R | R | R | R | S | S |
| WZZ4-15 | S | S | R | R | S | S | R | S | I | R | R | R | R | R |
| WZZ5-01 | S | S | R | R | R | S | S | S | R | R | R | S | R | R |
| WZZ5-02 | S | S | R | I | R | S | R | S | R | R | R | R | I | R |
| WZZ5-03 | S | S | R | S | S | S | R | S | R | R | R | I | R | R |
| WZZ5-04 | S | S | R | R | R | S | R | S | R | R | R | R | S | R |
| WZZ5-05 | S | S | R | S | S | S | R | R | R | R | R | R | S | R |
| WZZ5-06 | S | S | R | R | R | S | S | S | R | R | R | R | R | R |
| WZZ5-08 | S | S | R | I | R | S | R | S | R | R | R | R | I | R |
| WZZ5-09 | S | S | R | R | S | S | R | S | R | R | R | R | R | R |
| WZZ5-10 | S | S | R | S | R | S | R | S | R | R | R | R | S | S |
| WZZ5-11 | S | S | R | R | S | S | R | R | S | R | R | R | R | R |
| WZZ5-12 | S | S | R | I | R | S | R | S | R | R | R | S | R | R |
| WZZ5-13 | S | S | R | S | R | S | S | S | R | R | R | R | S | R |
| WZZ5-14 | S | S | R | R | R | S | R | R | R | R | R | R | R | R |
| WZZ5-15 | S | S | R | I | R | S | R | S | R | R | R | I | R | R |

Note: R=Resistant, I=Intermediate, S= Susceptible. P: Penicillin; AMC: Amoxicillin/ clavulanic acid; GM: Gentamicin; E: Erythromycin; TIL: Tilmicosin; FFC: Florfenicol; SIZ: Sulfisoxazole; TMP-SME: Trimethoprim/Sulfamethoxazole; VAN: Vancomycin; DOX: Doxycycline; TE: Tetracycline; ENR: Enrofloxacin; LZD: Linezolid; TIA: Tiamulin.

**Table S2 Minimal Inhibitory Concentration Breakpoints for *Enterococcus spp***

| Category | | | Antimicrobial  Agent | | Breakpoints（μg/mL） | | |
| --- | --- | --- | --- | --- | --- | --- | --- |
|  |  |  |  |  | S | I | R |
| Beta-lactams | | Penicillin (P) | | ≤8 | | — | ≥16 |
|  |  | Amoxicillin/ clavulanic acid (AMC) | | ≤ 8/4 | | 16/8 | ≥ 32/16 |
| Aminoglycosides | | Gentamicin (GM) | | ≤4 | | 8 | ≥16 |
| Macrolides | | Erythromycin (E) | | ≤0.5 | | 1-4 | ≥8 |
|  |  | Tilmicosin (TIL) | | ≤8 | | 16 | ≥32 |
| Phenicols | Florfenicol（FFC） | | ≤2 | | 4 | ≥8 |  |
| Sulfonamides | Sulfisoxazole（SIZ） | | ≤256 | | — | ≥512 |  |
|  | Trimethoprim/Sulfamethoxazole（TMP-SME） | | ≤ 2/38 | | — | ≥4/76 |  |
| Glycopeptides | | Vancomycin（VAN） | | ≤4 | | 8-16 | ≥32 |
| Tetracyclines | | Doxycycline（DOX） | | ≤4 | | 8 | ≥16 |
|  |  | Tetracycline（TE） | | ≤4 | | 8 | ≥16 |
| Quinolones | | Enrofloxacin（ENR） | | ≤0.5 | | 1-2 | ≥4 |
| Oxazolidinones | | Linezolid（LZD） | | ≤2 | | 4 | ≥8 |
| Pleuromutilins | | | Tiamulin（TIA） | ≤16 | | — | ≥32 |

**Table S3** **The primers of resistance genes**

| Target gene | Primer name | Primer sequence（5’- 3’） | Product length(bp) | | Annealing temperature (℃) | | References |
| --- | --- | --- | --- | --- | --- | --- | --- |
| *cfr* | *cfr*-F  *cfr*-R | TGAAGTATAAAGCAGGTTGGGAGT  ACCATATAATTGACCACAAGCAGC | | 974 | | 50 | (Yanhong, 2020) |
| *cfr(B)* | *cfr(B)*-F  *cfr(B)*-R | ACGGTTCTTCCTAAATCACT  TCAATAGAATCATTAACTCC | | 665 | | 45 | (Yanhong, 2020) |
| *optrA* | *optrA*-F  *optrA*-R | GCACCAGACCAATACGATACAA  TCCTTCTTAACCTTCTCCTTCTCA | | 750 | | 49 | (Mengyan et al., 2019) |
| *poxtA* | *poxtA*-F  *poxtA*-R | GGTCTGACTGGCTTGTTTTGCT  ATAAGGTCGGTATTGTCGGCGT | | 753 | | 53 | (Mengyan et al., 2019) |
| *fexA* | *fexA*-F  *fexA*-R | TTGGGAAGAATGGTTCAGGG  ATCGGCTCAGTAGCATCACG | | 977 | | 50 | (Yanhong, 2020) |
| *fexB* | *fexB*-F  *fexB*-R | GCAAGTTAGTTGGGTCGG  AAGCGATACCTATCCCTAAAC | | 786 | | 45 | (Yanhong, 2020) |
| *floR* | *floR*-F  *floR*-R | GCGATATTCATTACTTTGGC  TAGGATGAAGGTGAGGAATG | | 425 | | 45 | (Yi,2019) |
| *cml* | *cml*-F  *cml*-R | TAGGAAGCATCGGAACGTTGAT  CAGACCGAGCACGACTGTTG | | 665 | | 56 | (Yan,2013) |
| *lsa*(E) | *lsa*(E)-F  *lsa*(E)-R | TGTCAAATGGTGAGCAAACG  TGTAAAACGGCTTCCTGATG | | 486 | | 48 | (Yanhong, 2020) |

**Table S4 The primers of plasmid *rep* genes**

| *Rep* family | Target gene | | Primer sequence（5’- 3’） | Product length(bp) | Annealing temperature (℃) | References | |
| --- | --- | --- | --- | --- | --- | --- | --- |
| 1 | pIP501 | | CATTTACGCATTGGACAC  AGCCCTTATTATCTGTTTGT | 484 | 43 | (Jensen et al., 2010) | |
| 2 | pRE25 | | GAGAACCATCAAGGCGAAAT  ACCAGAATAAGCACTACGTACAATCT | 630 | 47 | (Jensen et al., 2010) | |
| 6 | pS86 | | TTCTTTGCGTTATGCGCACG  GCGGACAGCTCAAACCTACT | 314 | 52 | (Jensen et al., 2010) | |
| 8 | pAM373 | | AGTTGTACAGAAAGCCCCAAAC  AGAAGCACACAATGCTGTTGG | 193 | 50 | (Jensen et al., 2010) | |
| 9 | pCF10 | CCAACGACGCAAAAATTGCATAC  GCATCACACGACGCAAACAT | | 742 | 50 | (Jensen et al., 2010) |  |
| 18 | pEF418 | ACACCAGTCGAAATGAATTT  AGGAATATCAAGTAATTCATGAAGT | | 462 | 52 | (Jensen et al., 2010) |  |
| 4 | pMBB1 | ACTATGTCGTTGAGTCTAATGACT  AGCAAGATAGAATATTTACTTTTAAGTTT | | 430 | 52 | (Jensen et al., 2010) |  |
| 11 | pEF1071 | TCTAGAATGCGTAAAAAGG  CCTTTGAAGATWGCRGTWAG | | 500 | 52 | (Jensen et al., 2010) |  |
| 14 | pRI | GAAAGYTTRGATAGYTTTGC  RTTTTGRCTTTCTTSYTTCA | | 164 | 52 | (Jensen et al., 2010) |  |
| 17 | pRUM | TACTAACTGTTGGTAATTCGTTAAAT  ATCAAGGACTCAACCGTAATT | | 604 | 52 | (Jensen et al., 2010) |  |

**Table S5 Primers used for Multilocus Sequencing Typing of *E. faecalis***

| Target gene | Primer  name | | Primer sequence（5’**-** 3’） | Product length(bp) | References |
| --- | --- | --- | --- | --- | --- |
| ***gdh*** | *gdh*-F | GGCGCACTAAAAGATATGGT | | 530 | (Ruiz et al,2006) |
|  | *gdh*-R | CCAAGATTGGGCAACTTCGTCCCA | |  |  |
| ***gyd*** | *gyd*-F | CAAACTGCTTAGCTCCAATGGC | | 395 | (Ruiz et al,2006) |
|  | *gyd*-R | CATTTCGTTGTCATACCAAGC | |  |  |
| ***pstS*** | *gyd*-F | CGGAACAGGACTTTCGC | | 583 | (Ruiz et al,2006) |
|  | *gyd*-R | ATTTACATCACGTTCTACTTGC | |  |  |
| ***gki*** | *gki*-F | GATTTTGTGGGAATTGGTATGG | | 438 | (Ruiz et al,2006) |
|  | *gki*-R | ACCATTAAAGCAAAATGATCGC | |  |  |
| ***aroE*** | *aroE*-F | TGGAAAACTTTACGGAGACAGC | | 459 | (Ruiz et al,2006) |
|  | *aroE*-R | GTCCTGTCCATTGTTCAAAAGC | |  |  |
| ***xpt*** | *xpt*-F | AAAATGATGGCCGTGTATTAGG | | 456 | (Ruiz et al,2006) |
|  | *xpt*-R | AACGTCACCGTTCCTTCACTTA | |  |  |
| ***yiql*** | *yiql*-F | CAGCTTAAGTCAAGTAAGTGCCG | | 436 | (Ruiz et al,2006) |
|  | *yiql*-R | GAATATCCCTTCTGCTTGTGCT | |  |  |

**Table S6 MLST typing results of 30 *E. faecalis* strains**

| Isolates | Genotype | ST type |
| --- | --- | --- |
| NB116 | *poxtA* | 5 |
| HZ211 | *poxtA* | 16 |
| WZ208 | *poxtA* | 16 |
| JH217 | *optrA*+*poxtA* | 16 |
| WZ315 | *optrA* | 69 |
| NB304 | *optrA* | 75 |
| HZ115 | *poxtA* | 100 |
| JH316 | *optrA* | 116 |
| JH116 | *poxtA* | 116 |
| QZ211 | *optrA* | 167 |
| QZ513 | *optrA*+*poxtA* | 256 |
| QZ114 | *poxtA* | 287 |
| WZ319 | *poxtA* | 300 |
| WZ217 | *optrA* | 452 |
| QZ304 | *optrA* | 480 |
| NB217 | *optrA*+*poxtA* | 506 |
| JH107 | *optrA* | 506 |
| HZ318 | *optrA* | 506 |
| QZ119 | *optrA* | 618 |
| JH209 | *optrA* | 631 |
| WZ108 | *optrA* | 632 |
| QZ306 | *poxtA* | 632 |
| HZ406 | *optrA*+*poxtA* | 653 |
| HZ104 | *optrA* | 714 |
| HZ207 | *optrA* | 714 |
| NB102 | *optrA* | 714 |
| NB319 | *poxtA* | 714 |
| NB509 | *optrA* | 714 |
| JH321 | *poxtA* | 714 |
| WZ407 | *optrA*+*poxtA* | 727 |

**Table S7 Antimicrobial susceptibility test in *optrA* postive isolates, transconjugants and JH2-2**

| Isolates | ERN | VAN | TMP-SME | DOX | FFC | TIA | GM | LZD | TET |
| --- | --- | --- | --- | --- | --- | --- | --- | --- | --- |
| HZ104 | 512 | 0.125 | 0.06 | 16 | 64 | 128 | 4 | 2 | 128 |
| HZ104-JH2-2 | 512 | 0.125 | 16 | 16 | 32 | 64 | 2 | 2 | 128 |
| HZ207 | 512 | 0.125 | 0.06 | 8 | 32 | 256 | 1024 | 4 | 64 |
| HZ207-JH2-2 | 512 | 0.125 | 16 | 8 | 32 | 64 | 256 | 4 | 32 |
| HZ318 | 512 | 64 | 32 | 4 | 128 | 256 | 4096 | 16 | 128 |
| HZ318-JH2-2 | 512 | 2 | 16 | 2 | 32 | 256 | 2048 | 8 | 128 |
| HZ406 | 512 | 64 | 32 | 0.5 | 32 | 32 | 4096 | 8 | 128 |
| HZ406-JH2-2 | 256 | 16 | 32 | 1 | 16 | 32 | 2048 | 4 | 64 |
| QZ119 | 512 | 32 | 32 | 1 | 32 | 8 | 4096 | 2 | 128 |
| QZ119-JH2-2 | 1 | 0.125 | 16 | 1 | 8 | 8 | 1 | 2 | 128 |
| QZ211 | 512 | 1 | 32 | 32 | 512 | 16 | 4096 | 8 | 128 |
| QZ211-JH2-2 | 2 | 1 | 16 | 4 | 128 | 8 | 1024 | 4 | 128 |
| QZ304 | 512 | 1 | 0.5 | 32 | 64 | 128 | 8 | 8 | 128 |
| QZ304-JH2-2 | 32 | 0.5 | 16 | 4 | 32 | 64 | 4 | 4 | 64 |
| NB304 | 4 | 1 | 2 | 8 | 32 | 16 | 8 | 8 | 8 |
| NB304-JH2-2 | 1 | 0.125 | 16 | 1 | 16 | 8 | 1 | 4 | 8 |
| NB409 | 512 | 1 | 0.06 | 32 | 32 | 256 | 8 | 4 | 128 |
| NB409-JH2-2 | 64 | 0.25 | 16 | 2 | 16 | 8 | 8 | 2 | 128 |
| JH108 | 512 | 1 | 0.06 | 32 | 32 | 128 | 2048 | 2 | 128 |
| JH108-JH2-2 | 32 | 0.25 | 16 | 4 | 8 | 64 | 512 | 2 | 64 |
| JH214 | 512 | 0.5 | 0.06 | 32 | 64 | 256 | 512 | 8 | 128 |
| JH214-JH2-2 | 64 | 0.125 | 16 | 4 | 16 | 64 | 64 | 4 | 64 |
| WZ107 | 512 | 0.5 | 32 | 8 | 128 | 256 | 64 | 8 | 8 |
| WZ107-JH2-2 | 128 | 0.5 | 32 | 1 | 64 | 128 | 32 | 4 | 2 |
| JH2-2 | 1 | 0.125 | 16 | 1 | 4 | 8 | 1 | 2 | 1 |

**References**

Jensen L, Garcia L, Valenzula A, et al. (2010). A classification system for plasmids from *Enterococci* and other Gram-positive bacteria. Journal of Microbiology Methods, 80(1): 25-43.

Mengyan Y, Runhao Y, Nannan W, et al. (2019). Analysis of Prevalence of Amide Alcohol-oxazolidinone Cross－resistance Genes in A Large－scale Swine Farm. Acta Agriculture Jiangxi, 31 (10):116-121. DOI: 10.19386/j.cnki.jxnyxb. 2019.10.20.

Yan C, Zhixun Y, Yuhong Z, et al. (2013). Resistance analysis of *Salmonella* Indiana to the chlmphenicols. Microbiology China, 040(007): 1225-30. Doi: 10.13344 /j.microbiol.china.2013.07.008.

Yanhong S. (2020). The epidemiological characteristics and dissemination mechanism of resistance gene *optrA* and *lsa*(E) among *enterococcus* and *streptococcus suis* from swine. Northwest A&F University. DOI: 10.27409/d.cnki.gxbnu. 2019.000055.

Yi G. (2019). Antimicrobial susceptibility and the epidemiological characteristics of amphenicols/oxazolidinones and fosfomycin resistance genes in *Enterococcus* from swine. Nanjing Agricultural University. DOI: 10.27244/d.cnki.gnjnu. 2019.001749.

Ruiz G.P., Bonten M.J., Robinson D.A., et al. (2006). Multilocus sequence typing scheme for *Enterococcus faecalis* reveals hospital-adapted genetic complexes in a background of high rates of recombination. Journal of Clinical Microbiology, 44(6):2220-8. Doi: 10.1128/JCM.02596-05.
